# Cyclic AMP Represents a Crucial Component of Treg Cell-Mediated Immune Regulation

**DOI:** 10.3389/fimmu.2016.00315

**Published:** 2016-08-29

**Authors:** Matthias Klein, Tobias Bopp

**Affiliations:** ^1^University Medical Center, Institute for Immunology, Johannes Gutenberg-University, Mainz, Germany

**Keywords:** cyclic AMP, regulatory T cells, FOXP3, adenosine, immune regulation, immune tolerance network, suppression

## Abstract

T regulatory (Treg) cells are one of the key players in the immune tolerance network, and a plethora of manuscripts have described their development and function in the course of the last two decades. Nevertheless, it is still a matter of debate as to which mechanisms and agents are employed by Treg cells, providing the basis of their suppressive potency. One of the important candidates is cyclic AMP (cAMP), which is long known as a potent suppressor at least of T cell activation and function. While this suppressive function by itself is widely accepted, the source and the mechanism of action of cAMP are less clear, and a multitude of seemingly contradictory data allow for, in principle, two different scenarios of cAMP-mediated suppression. In one scenario, Treg cells contain high amounts of cAMP and convey this small molecule *via* gap junction intercellular communication directly to the effector T cells (Teff) leading to their suppression. Alternatively, it was shown that Treg cells represent the origin of considerable amounts of adenosine, which trigger the adenylate cyclases in Teff cells *via* A_2A_ and A_2B_ receptors, thus strongly increasing intracellular cAMP. This review will present and discuss initial findings and recent developments concerning the function of cAMP for Treg cells and its impact on immune regulation.

## Introduction

The immune system commands an impressive armament to repel invading microorganisms. The overwhelming part of such an antimicrobial immune response is coordinated and initiated by CD4^+^ T helper (Th) cells, which recognize foreign antigens through their T cell receptor after presentation by antigen-presenting cells. A huge Th cell receptor repertoire ensures that almost all pathogens can be recognized and subsequently be eliminated. However, this virtually exhaustless T cell receptor repertoire obviously bears the risk of containing autoaggressive T cells (1). This was demonstrated by the transfer of naive Th cells, which represent approximately 90% of all peripheral CD4^+^ Th cells into T cell-deficient mice that led to a multitude of autoimmune diseases indicating that a considerable part of this Th cell population is potentially autoreactive (2–4). These potentially autoreactive naive Th cells are usually kept in check when CD25^+^ peripheral Th cells are cotransferred together with CD25^−^ T cells, thus indicating that this residual T cell compartment that was separated from naive T cells before transfer is capable to prevent autoimmune symptoms (5, 6). Today, we know that this suppressive Th cell population mainly consists of T regulatory (Treg) cells which were found to control T effector (Teff) cells *in vitro* in a contact-dependent manner. The Treg/Teff cell interaction was shown to suppress preferentially IL-2 production and proliferation of the Teff cells – a hallmark of clonal T cell expansion (7). Concerning the suppressive mechanism(s), the usage of cytokine-deficient and cytokine receptor-deficient mice could exclude that IL-10 and TGF-β – at least *in vitro* – mediated the suppressive properties of Treg cells (7, 8). Subsequently, the characterization of the transcription factor forkhead box protein 3 (FOXP3), as a lineage-specific marker for Treg cells, and the generation of FOXP3 reporter mice strongly boosted Treg cell research. Continuative analyses revealed that FOXP3 is crucial for Treg cell development and function (9–11). These findings provided the opportunity to screen the FOXP3-regulated Treg cell transcriptome which revealed that the expression of a cyclic AMP (cAMP)-degrading phosphodiesterase (PDE3b) is strongly repressed in Treg cells, whereas the expression of ectonucleotidases (CD39 and CD73) as well as expression of adenylyl cyclase 9 (AC9), an enzyme promoting *de novo* generation of intracellular cAMP, was upregulated (12, 13). Therefore, reduced expression of phosphodiesterase (PDE3b) implied a decreased degradation of intracellular cAMP accompanied by a strong production of cAMP due to strong expression of AC9, while elevated expression of CD39/CD73 should lead to an increased generation of extracellular adenosine in the proximity of Treg cells. Hence, FOXP3-dependent transcriptional profiling suggested that the suppressive properties of Treg cells is based at least partially on comparatively high amounts of intracellular cAMP concomitantly with an enhanced ability to generate extracellular adenosine from adenosine triphosphate (ATP) [reviewed in Ref. (14, 15)].

## Intracellular cAMP Enables Treg Cells to Maintain the Balance of the Immune Tolerance Network During Immune Homeostasis

Intracellular cAMP has long been recognized as a potent inhibitor of T cell activation. Especially, agents that elevated cAMP in T cells like cholera toxin, prostaglandin E2, and forskolin were found to strongly impair IL-2 production and T cell proliferation (16–19). Comparative analyses of intracellular cAMP revealed that Treg cells contained high intracellular amounts of cAMP, while it was hardly detectable in Teff cells (20). In addition, co-activation of cocultured Treg and Teff cells resulted in a considerable intracellular increase of cAMP in Teff cells, suggesting a cell contact-dependent transfer of cAMP. One possibility for cell contact-dependent transfer was gap junction intercellular communication (GJIC). GJIC was demonstrated by employing the fluorescent dye calcein which can only be transferred between T cells by gap junctions (21, 22). The functional consequence of such a GJIC-mediated transfer of cAMP between Treg and Teff cells was a strong reduction of IL-2 expression, and as a consequence, inhibition of proliferation, which was both reversed in the presence of the GJIC inhibitor GAP27. In addition, it was shown that the coculture of murine Treg cells and dendritic cells (DC) led to a strong elevation of cAMP in DC concomitantly with an immediate downregulation of CD80/CD86 costimulators (23). This Treg cell-mediated suppression of DC activation *via* transfer of cAMP was suggested to be decisively involved in the control of a Graft-versus-host disease (GvHD) by Treg cells. Accordingly, the potency of Treg cells to ameliorate a GvHD was found to be strongly increased in the presence of PDE-inhibitors like rolipram (24). In agreement with these findings, it was shown that neonatal human Treg cells suppress DC activation by CTLA-4 and cAMP (25).

The importance of GJIC for Treg-mediated suppression was recently emphasized by the finding that diabetic NOD mice showed an impaired expression of connexin 43 (Cx43), an important component of gap junctions, leading in consequence to a strongly reduced suppressive potency of their Treg cells (26). Overexpression of Cx43 in such Treg cells increased their suppressive properties roughly to the level of non-diabetic young NOD mice. The same outcome could be observed when Cx43-mediated GJIC was strengthened *via* the treatment with alpha-connexin carboxyl-terminal peptide 1 (αCT-1). αCT-1 is a unique synthetic peptide that mimics a cytoplasmic regulatory domain of Cx43 and that specifically disrupts the interaction between Cx43 und its binding partner zonula occludens (ZO)-1, ultimately leading to an enhanced gap junction aggregation (27, 28). However, treatment with αCT-1 failed when Cx43-deficient Treg cells were used, indicating the specificity of action of this agent (26).

The comparatively high content of cAMP in Treg cells was described to result from a strong FOXP3-mediated reduction of PDE3b expression concomitantly with a considerable suppressive activity of this T cell type (29). Further analyses of the underlying molecular mechanisms revealed that FOXP3 not only binds and regulates the *Pde3b* locus but also additionally downregulates expression of the miRNA miR-142-3p, a potent inhibitor of AC9, which catalyzes the conversion of ATP to 3′,5′-cAMP. As a consequence of the impaired targeting of AC9 mRNA due to reduced miR-142-3p expression in Treg cells, these cells contain high levels of AC9, driving elevated intracellular cAMP level. Accordingly, transfection of Treg cells with miR-142-3p not only reduced AC9 expression and intracellular cAMP levels but also considerably impaired the suppressive properties of these Treg cells. The same effect was observed when Treg cells were transfected with AC9 siRNA, again preventing *de novo* generation of intracellular cAMP by AC9. Thus, FOXP3 strongly promotes AC9-dependent intracellular cAMP production by suppressing expression of miR-142-3p and PDE3b as well. Concerning human Treg cells, it was shown that polyclonal activation as well as activation *via* CD4, known to induce tolerance, strongly increased the concentration of intracellular cAMP. The irreversible blockade of adenylate cyclases (AC) by MDL12 inhibited this upregulation of cAMP concomitantly with a strong impairment of their suppressive potency – *in vitro* as well as *in vivo* – and led to an increased proliferation of these AC-blocked Treg cells (30). Nevertheless, such Treg cells regained their suppressive properties after several days of expansion probably as a consequence of the dilution of the AC inhibitor MDL12. Similar to MDL12, the ectopic expression of cAMP-degrading phosphodiesterase, PDE4c, also prevented the increase of intracellular cAMP in human Treg cells and strongly impaired their suppressive capacity. By contrast, it could be shown that the inhibition of PDE4 by rolipram strongly enhanced the suppressive potency of Treg cells *in vitro* and *in vivo*. Transfer of rolipram-treated Treg cells strongly inhibited asthma symptoms in a prophylactic as well as a therapeutic preclinical animal model of experimental asthma (31). As a consequence of Treg–Th2 interaction *in vivo*, a strong increase of intracellular cAMP could be observed in lung-resident Th2 cells. Additional studies revealed that asthma-promoting Th1 and Th2 cells exhibited a similar increase of intracellular cAMP after contact with Treg cells *in vitro* and *in vivo*, but that Th1 cells were far more sensitive to Treg-mediated suppression (32). This was in agreement with the finding that comparatively high cAMP contents are needed to strongly inhibit Th2-derived IL-4 production, while comparatively low cAMP concentrations are sufficient in suppressing IL-2 production and subsequent proliferation of Th1 cells (18).

## Extracellular Adenosine is Required to Limit a Potentially Self-Destructive Inflammatory Immune Response

Inflammatory immune reactions are accompanied by the release of high amounts of ATP in the extracellular space where it is converted into AMP by CD39 and dephosphorylated to adenosine by CD73 (33). Adenosine has strong anti-inflammatory influence on immune cells preferentially by triggering A_2A_ and A_2B_ receptors leading to the generation of intracellular cAMP. Interestingly, the anti-inflammatory action of adenosine is exploited by tumors where adenosine was shown to accumulate, thereby preventing immunological tumor regression (34, 35). The tumor-protective role of adenosine was underlined using mice deficient for the genes *Adora2a*, encoding for A_2A_ or *Adora2b*, encoding for A_2B_ receptors which show enhanced elimination of tumors compared to A_2A_ and A_2B_ receptor sufficient wild-type mice (35–37). Among mouse T cells, CD39 and CD73 were described to be preferentially co-expressed in Treg cells (38). This was in agreement with the finding that the expression of *Entpd1* (CD39) and *Nt5e* (CD73) was shown to be upregulated by FOXP3 (12). In line with this, inhibitors of CD39 and CD73 decreased the suppressive capacity of Tregs (39–41). Conclusively, *Entpd1*-deficient (CD39-deficient) Treg cells exhibited impaired suppressive properties *in vitro* in a coculture assay as well as *in vivo* using an allogeneic skin graft model upon cotransfer of Treg/Teff cells indicating the importance of this ectonucleotidase for the suppressive function of Treg cells. Similarly, in a model of acute lung injury (ALI), adoptive transfer of *Nt5e*-deficient (CD73-deficient) Treg cells failed to resolve ALI adequately, whereas transfer of wild-type Treg cells led to normal resolution (42). Furthermore, it was shown in a model of acute kidney injury that DC treated with an A_2A_ receptor agonist could protect the kidney by suppressing DC-dependent NKT cell activation, suggesting a strong immunosuppressive influence of adenosine on the accessory functions of such DC (43).

Interestingly, Teff cells that represent a major target of Treg cell suppressive activity were found to express the T cell-typical adenosine receptor A_2A_ not before day 4 after activation, thus excluding immediate early suppressive influence by this mechanism (38). Accordingly, the authors stated that the adenosine-driven suppression was most probably functional during the late phases of Teff cell activation. Nonetheless, the importance of A_2A_ receptors on Teff cells for Treg cell-mediated suppression in the late phases of an adaptive immune response *in vivo* was demonstrated in an adoptive T cell transfer model of chronic colitis. Herein, wild-type colitogenic CD4^+^ Teff cells were considerably suppressed after cotransfer of Treg cells in SCID mice, whereas their *Adora2a* (A_2A_ receptor)-deficient counterparts could not be inhibited by the cotransferred Treg cells so that the transfer of such Teff cells led to the development of colitis (44). In addition, and in line with the aforementioned results, A_2A_ receptor-deficient Treg cells could not prevent colitis caused by pathogenic wild-type Teff cells, suggesting that adenosine is also of central importance for the suppressive function of Treg cells in this model of inflammatory bowel disease. Hence, it can be reasonably assumed that in addition to reduced PDE3b expression and enhanced AC9 expression, high intracellular levels of cAMP in Treg cells also depend on adenosine-driven elevation of cAMP. This assumption was corroborated by a recent publication of Sitkovsky’s group demonstrating that adenosine stimulated the expansion of Treg cells and concomitantly raised their suppressive capacity (45). Thus, adenosine can directly inhibit Teff cell activation and simultaneously improve the suppressive function of the Treg cell compartment most possibly by affecting cAMP production.

**Figure 1:**
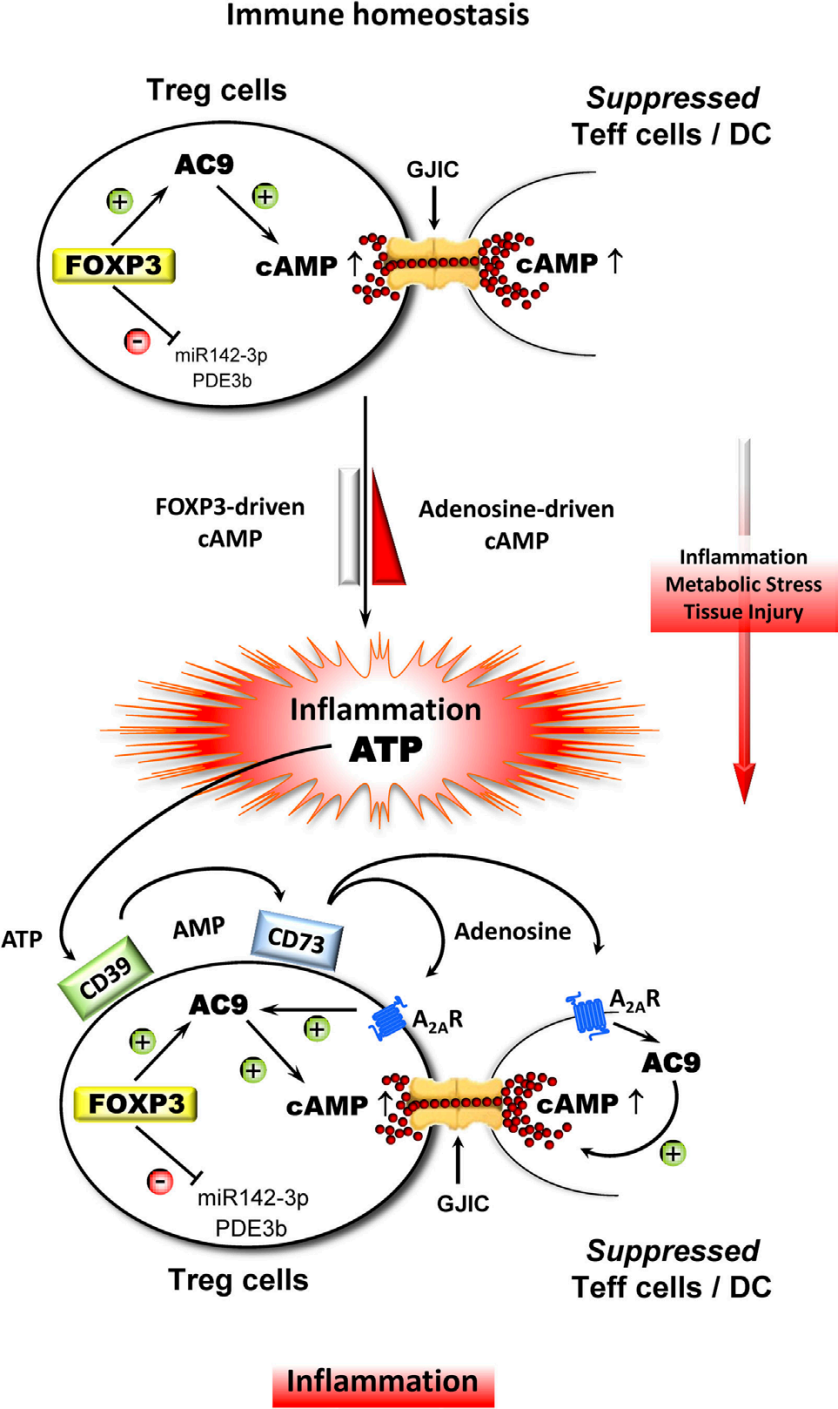
**Adenosine strongly improves the suppressive influence of Treg cell-derived cAMP in the course of inflammation**. During immune homeostasis, Treg cells stabilize the ITN with the aid of endogenous cAMP that is driven by FOXP3, which indirectly upregulates adenylate cylase 9 (AC9) through the inhibition of miR142-3p and which concomitantly downregulates cAMP-degrading phosphodiesterase 3b (PDE3b). As a result, Treg cells contain comparatively high amounts of cAMP leading to the suppression of Teff cells and DC *via* gap junctional intercellular communication (GJIC). Inflammation in combination with metabolic stress and tissue injury results in a massive release of ATP, which represents a powerful danger signal that serves as an additional local inflammatory booster that bears the risk of collateral damage by uncontrollable immune reactions. Therefore, metabolization of ATP by ectonucleotidases CD39 and CD73 that leads to increased local amounts of adenosine prevents such a fatal development especially through triggering the adenosine receptor A_2A_ (A_2A_R) on Treg and Teff cells and DC as well. The A_2A_R-mediated elevation of intracellular cAMP inhibits the activation of Teff cells and impairs the accessory function of DC and simultaneously strongly improves the suppressive activity of Treg cells.

In conclusion, high intracellular cAMP levels in Treg cells provide a major contribution to a balanced immune tolerance network (ITN) in the course of immune homeostasis when Treg cells are preferentially stimulated *via* the T cell receptor complex in combination with rather low costimulatory influences (Figure 1). However, in the course of a local inflammation which is additionally boosted by a myriad of potent microorganism-derived costimulators (bacterial, viral, fungal), an immune response can easily get out of control leading to collateral damage and with that to detrimental consequences. To prevent such a fatal scenario, the suppressive capacity of Treg cells can obviously be strongly improved by adenosine which originates from the CD39/CD73-mediated degradation of extracellular ATP that is characteristically released in huge amounts during such collateral damage of inflamed tissue. This ATP-derived adenosine profoundly increased the overall cAMP content of the involved cells as well as the suppressive arsenal of the local Treg cell compartment which is automatically reduced when inflammation is resolved enabling the ITN to return to immune homeostasis. Thus, the ITN follows an escalating suppressive strategy that allows for the initiation of an effective immune response by breaking a relatively weak suppression that is based on intracellular cAMP which subsequently exploits the locally originating ATP leading to the generation of immune suppressive adenosine to further strengthen intracellular cAMP levels, and by that, curbing a potentially life threatening immune reaction.

## Author Contributions

All authors listed have made substantial, direct, and intellectual contribution to the work and approved it for publication.

## Conflict of Interest Statement

The authors declare that the research was conducted in the absence of any commercial or financial relationships that could be construed as a potential conflict of interest.
